# Virtual reality reduces pain in palliative care–A feasibility trial

**DOI:** 10.1186/s12904-022-01058-4

**Published:** 2022-10-05

**Authors:** Miriam Guenther, Dennis Görlich, Florian Bernhardt, Esther Pogatzki-Zahn, Burkhard Dasch, Janina Krueger, Philipp Lenz

**Affiliations:** 1 Department of Palliative Care, University Hospital Muenster, Muenster, Germany; 2 Institute of Biostatistics and Clinical Research, University of Muenster, Muenster, Germany; 3 Department of Anesthesiology, Intensive Care and Pain Medicine, University Hospital Muenster, Muenster, Germany; 4 Specialized Outpatient Palliative Care Service Muenster, Muenster, Germany; 5 Department of Palliative Care, University of Muenster, Albert-Schweitzer-Campus 1, Building W 30, D-48149 Muenster, Germany

**Keywords:** Palliative care, Virtual reality, Pain control, Pain treatment

## Abstract

**Background:**

Effective symptom control is a stated goal of palliative care (PC) to improve quality of life for terminally ill patients. Virtual reality (VR) provides temporary escapes from pharmacologically resistant pain and allows for experiences and journeys patients may not access in any other way. Enabling wishes through virtual worlds may also offer additional benefits such as controlling psychological and physical symptoms.

**Aims:**

We investigated the feasibility of a single VR experience as a viable, satisfying, and effective tool for end-of-life pain relief for inpatients presenting palliative needs.

**Design:**

This is an observational, single-arm and national single-center feasibility trial.

**Methods:**

A one-time VR experience with a selection of several videos and games was offered to 45 inpatients receiving PC at Muenster University Hospital. Patients with brain tumors, brain metastases, seizures, motion sickness, claustrophobia, vertigo, hearing or visual impairment, or unable to consent were excluded. Primary outcome measured patient reported pain on a visual analogue scale (VAS). We also measured Karnofsky performance status, health-related quality of life (HRQOL) using the EQ-5D-5 L questionnaire, and the Pain Out Questionnaire for postoperative pain.

**Results:**

We analyzed data from 21 women (52.5%) and 19 men (47.5%) at an average age of 51.9 (SD: 15.81) years. The mean Karnofsky score among the sample was 45.5 (SD: 14.97) and the HRQOL was 41.9 (SD: 23.08). While no serious side effects were reported during the intervention, three patients experienced nausea (7%), two headaches (5%), and three reported dry eyes (7%) afterwards. Significant pain reduction (baseline VAS 2.25 (SD: 0.4399)) was demonstrated during (VAS 0.7 (SD: 0.2983, p < 0.0001)), immediately after (VAS 0.9 (SD: 0.3354, p = 0.0001)) and one hour after the intervention (VAS 1.15 (SD: 0.4163, p = 0.0004)). More than 80% rated the VR experience as very good or good (85%, n = 34) and intended to make use of the device again (82.5%, n = 33). However, two participants (5%) also expressed sadness by becoming aware of old memories and previous opportunities that are gone.

**Discussion:**

The present pilot study suggests that VR seems to be a feasible and effective tool for pain relief in PC. Its use encompasses the approach of a total pain and symptom therapy and enhances patients’ dignity and autonomy. Future research ought to include if and to what extent VR could reduce the necessity of pharmacological pain relief.

## Introduction

The World Health Organization defines palliative care (PC) as an interdisciplinary specialty that aims to improve the lives of patients suffering from life-threatening disease and is most commonly provided by PC consultation teams or PC units in inpatient healthcare services. There is evidence that PC is associated with lower symptom burden, improved quality of life, and prolonged survival [1–4].

A comprehensive concept is required whenever seriously ill and dying people are accompanied, treated and counselled, in order to cope with life-limiting diseases, usually associated with various ailments and high symptom burden during their progression [5, 6]. Besides physical deterioration, psychosocial symptoms such as depression and loneliness can exacerbate the situation [7, 8]. As a result, also non-medical interventions, such as dignity therapy, are of great importance in PC [9].

Patients with advanced malignancies represent the largest group of people receiving hospice and palliative care. Hess et al. determined that only 8.1% of their sample had non-cancer diagnoses [10]. Most frequent symptoms of patients in PC with advanced cancer include fatigue, pain, dry mouth, anorexia, loss of weight and sleep problems [11]. However, according to See et al., the symptom burden between patients with malignant and non-malignant diseases is very similar after adjusting for confounders[12]. Among these patients, 71% are suffering from pain [13]. The proportion of PC patients suffering from pain is quantified to be 64% according to another survey [14]. Looking at inpatients who were treated by the Palliative Care Consultation Service (PCCS) at Muenster University Hospital between May 1, 2015 and May 31, 2016, 56.7% (278 of 490) were affected by pain (measured with visual analog scale, mean 5.1, SD 2.7, median 5.0) [15]. Observational research confirms that many patients suffering from cancer experience moderate or severe pain yet do not receive appropriate treatment [13, 16]. In 2022, Deandra et al. outlined in their review that still about 40% of patients with pain due to cancer are undertreated [17].

Increasing attention has been paid to non-pharmacological methods in the treatment of both acute and chronic pain in recent years. In fact, pain perception can be reduced by stimuli that attract attention [18]. Accordingly, it is hypothesized that virtual reality (VR) might help people to distract from painful stimuli [19, 20].

VR is a three-dimensional computer-generated 360° immersion provided by head-mounted displays or stereoscopic glasses. Paired with headphones and optional haptic feedback, the system creates a multi-sensory experience. As a result, perfect distraction and immersion in the “virtual world” can be created. Besides animated clips, 360° cameras also offer the technology for generating virtual impressions of any existing places. Other characteristics include real-time vision transmission, 3D interactions, and changes in the virtual environment following head movements.

Pain reduction through VR was already demonstrated in several studies [20–22]. It is considered that experiencing virtual worlds and environments necessitates sufficient mental capacity for distracting pain [19, 20]. Numerous studies evaluated its practical applicability in clinical settings [22–24]. Mallari et al. reviewed the existing research on the treatment of acute and chronic pain using VR. It was concluded that VR is an effective treatment for acute pain, although lacking any long-term benefit addressing chronic pain [25]. Feasibility studies showed high acceptance among different groups of participants and suggested further research [26–28]. In addition to the analgesia effects of VR, beneficial outcomes were demonstrated for anxiety, affect, and happiness. Since VR increases affect and fun, it may allow temporary escapes from isolation and depressing ambiences. Moreover, unpleasant or painful procedures tended to be more tolerable while experiencing VR [29].

Aim of this pilot study was to investigate the feasibility of VR for symptom control, in particular for the relief of pain in PC. We were also interested in the acceptability of a VR intervention for patients receiving PC and any adverse side effects.

## Patients and methods

### Study design

This study is an observational, single-arm and national single-center pilot study, performed at the University Hospital Muenster, Germany.

Each participant received a single VR intervention and was followed up for one hour regarding pain and further related parameters.

### Setting

The study was conducted at Muenster University Hospital, which is an urban tertiary care hospital providing healthcare services to a large catchment area. Our sample included inpatients suffering pain who were treated by the PCCS or received usual PC. Screening was performed by PCCS physicians, assisted by nurses specialized in PC. General information, informed consent, and the VR intervention as well as all questionnaires were given and answered in the patient’s rooms. Any patients unable to complete the documents unassisted received help from the medical student. Depending on their individual preferences and physical conditions, patients either were lying in bed or were sitting in a chair throughout their VR session. Patients were observed by a medical student both during the intervention and, as a safety precaution, for half an hour following the intervention to provide immediate medical action for any side effects. A PCCS team member collected the VR questionnaire within the same day or the day after intervention. Each patient was kept under the care of the PCCS until being discharged from hospital.

Due to our study design, no further follow-up was performed, even though we were able to exclude any negative long-term effects by continuing PC treatment.

Recruitment period started in September 2018 and was completed in May 2019.

### Participants

Informed consent was obtained from all patients. The study protocol conformed to the ethical guidelines of the 1975 Declaration of Helsinki and was a priori approved by the local ethics committee of the University of Muenster (2018-168-f-s). In addition, the study was registered (ClinicalTrials.gov Identifier: NCT03698526).

The feasibility trial includes data from 45 patients with advanced life-limiting and progressive diseases. Biostatistical consultation with case number planning prior to the study indicated at least 39–40 patients for an 80% power with a 5% drop-out rate. We solely included patients with the main problem pain. Except one patient, all participants were treated as inpatients at Muenster University Hospital with co-care by the PCCS or received usual PC. No threshold was set for pain level since our main goal was to evaluate an overall reduction. Thus, all patients suffering from pain were offered the opportunity to participate in the VR experience. Main complexity was coordinating screening by the outpatient PC service and visiting patients due to scheduling conflicts.

Inclusion criteria were: a minimum age of 18 years, progressive life-limiting disease in need of PC, and capacity to adequately understand the information and to give written consent in case of agreement. We excluded patients lacking the capacity to consent and those with hearing or visual impairments. Considering that there is a small presumed risk of VR induced seizures,[30] patients who had brain tumors, brain metastases, or a previous seizures were also excluded. Additionally, people who suffered from claustrophobia or vertigo were similarly rejected. All patients were asked about these symptoms verbally prior to study inclusion. This was based on the manufacturer’s information that using these devices may cause discomfort in people prone to motion sickness [30]. All patients were treated with long-acting analgesics based on their personal needs if necessary. It was ensured that no breakthrough pain medication was taken throughout the day the VR intervention was conducted.

### Intervention/VR technique

**Fig. 1:**
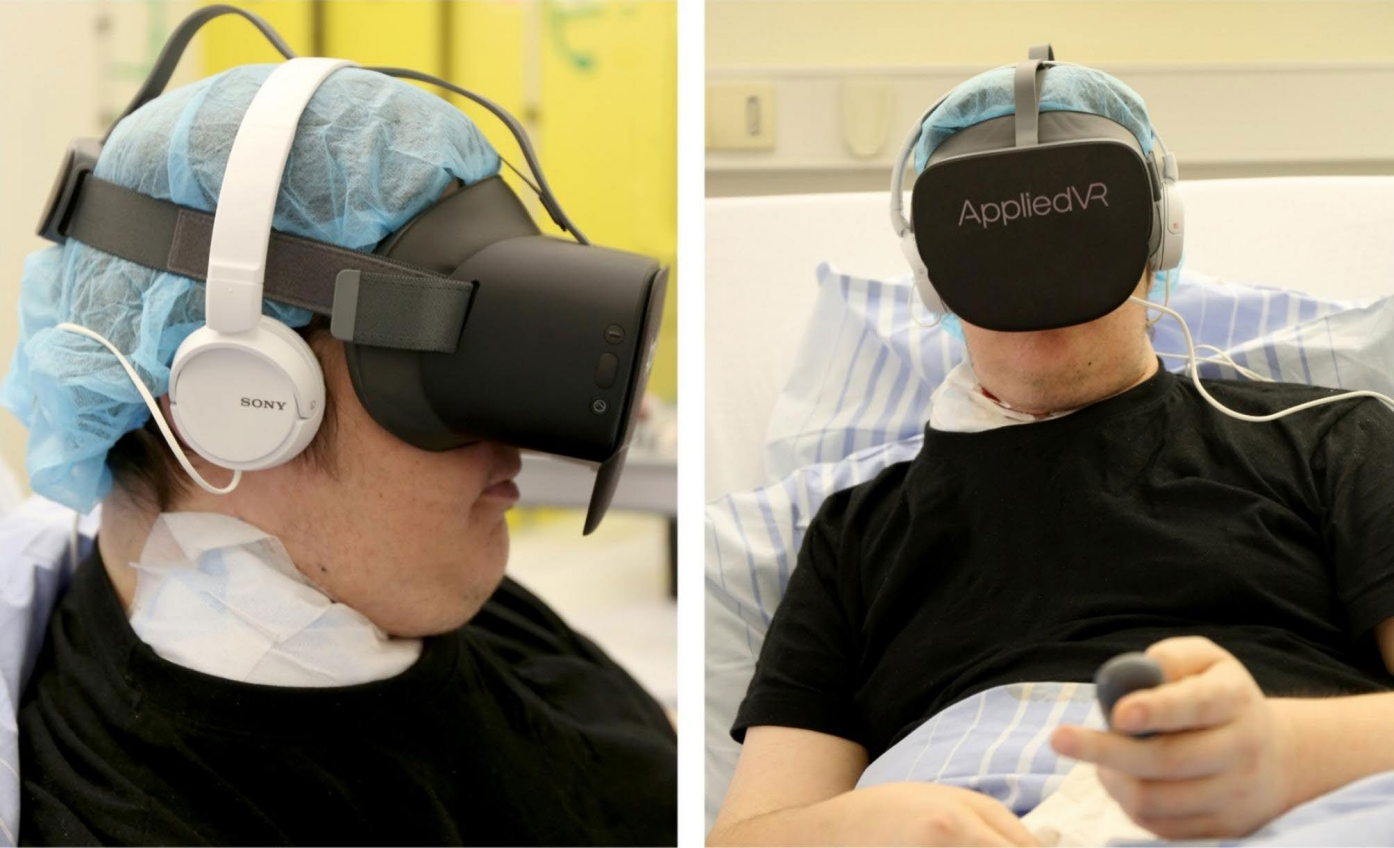
37-year-old patient suffering from metastatic malignant melanoma. A deep ulcerated wound caused massive pain requiring patient-controlled analgesia with hydromorphone. Due to bleeding, dressing needed to be changed twice a day. The patient was introduced to virtual reality for pain relief and relaxation purposes

We chose the Samsung Gear VR and PICO G2 4 K VR offered by AppliedVR (AppliedVR, 16,760 Stagg St Unit 216, Van Nuys, CA 91,406, USA, https://www.appliedvr.io/). These devices were explicitly developed for medical use and suitable in clinical settings (e.g. [21, 22, 27]). Samsung Gear VR consists of a head-mounted display (HMD) that has to be connected to a Samsung Galaxy S7, whereas PICO G2 4 K VR has a built-in screen (Fig. 1). Motion sensors allow the user to control their devices via head movements. As a result, virtual icons appear for selecting between various modes and menu items. A variety of immersive 360-degree videos for relaxation, distraction or escaping reality, and games are provided in the used app. Selection was dependent on personal preferences and individual needs. Videos are divided into three categories: journeys (Iceland and London), relaxation (various beaches and secluded places, Tibetan singing bowls, meditation) and animals (Dolphins Healing, Seal Hospital, Wild West, Farm Sanctuary).

For sanitary cleanliness, the devices were disinfected both before and after any use, including headsets and cell phones. In case of isolated patients, the foam cushioning has been changed.

### Variables

As primary outcome, patients’ self-reported pain intensity was measured using a visual analog scale (VAS). The patients marked their pain intensity on a horizontal line of 100 mm length, ranging from “no pain” (0 mm) to “very severe pain” (100 mm).

Based on our hypothesis that VR leads to significant pain reduction, we evaluated pain levels across multiple time points (pre, during, immediately after, and 1 h after the intervention). In addition, we collected a number of patient characteristics and procedural data prior to and after the VR experience.

The questionnaire used immediately after the intervention was particularly related to the pain during their VR experience. All of the following questions focused on the intensity of pain after the VR intervention. To analyze the VAS scores, each marked value was measured in millimeters and then converted into centimeters with one decimal place (e.g., if the mark was set at 52 mm, this corresponds to a value of 5.2).

Characterization of symptom-related disability at the day of the intervention was based on Karnofsky performance status scale. For the assessment of health-related quality of life (HRQOL), patients completed EQ-5D-5 L health questionnaire [31]. Both scores extend from 100 to 0, with low numbers corresponding to worse conditions. Prior to the intervention, patients stated whether they are able to understand English, have had previous experiences with VR or know about its use in healthcare. We also verbally investigated side effects caused by the VR intervention, such as dry eyes, headache, dizziness, or drowsiness. In addition, we asked patients whether they felt happiness or sadness throughout the intervention, how they rated their experience based on an ordinal scale (1–4, very good, good, satisfactory, or poor), and asked if they were interested in repeating any VR intervention.

To detect any differences between the perception of pain pre and post VR intervention, participants completed the Pain Out questionnaire at both times. Although this questionnaire is regularly used to measure patients’ satisfaction with postoperative pain management[32], we utilized it to collect data at different time points. For our research, this questionnaire provided some interesting supplementary information since it included questions concerning acute pain as well as questions related to the presence and duration of chronic pain[33]. Its core measure is the International Pain Outcomes Questionnaire [34], which primarily works with 11-item (0–10) numeric rating scales (NRS, higher numbers represent more severe symptoms) and binary items.

### Statistical and qualitative analysis

Descriptive statistics were used to analyze demographic data. We summarized continuous variables mainly by the mean and standard deviation (SD) or in some cases by median if specified. Categorical variables are presented as absolute and relative frequencies. Tests for normal distribution of pain scores measured by VAS were performed with Kolmogorov-Smirnov and Shapiro-Wilk tests. Continuous parameters were analyzed using the Wilcoxon-Mann-Whitney-Test. Two-sided p-values of ≤ 0.05 were considered statistically significant. Spearman’s Correlation was used to identify any correlation between parameters. This takes on values ranging from − 1 in the case of a negative to + 1 in the case of a positive correlation.

Statistical analyses were performed using SPSS Software (IBM Corp. Released 2017. IBM SPSS Statistics for Mac, Version 25.0. Armonk, NY: IBM Corp.) and *SAS Software (Version 9.4, SAS Institute Inc., Cary, NC, USA).*

## Results

### Patients characteristics

Our cohort included 45 patients (n = 45). We excluded four participants. Either they met exclusion criteria, or their exclusion was due to acute health problems or health deterioration that inhibited participation. We also excluded a patient who was receiving hospice care. Of the remaining 40 patients (89%) included, 37 (92.5%) received specialized PC provided by the PCCS, compared to 3 patients (7.5%) receiving usual PC. Gender balance was fairly equal, as 21 patients (52,5%) were female and 19 male (47,5%). Mean age was 51.9 ± 15.81 years, with the youngest patient being 19 years and the oldest 80 years old.

Albeit pain was the main problem for all patients when VR intervention was performed, initial contact with specialized PC resulted due to pain (57.5%, n = 23), organization of outpatient care (35%, n = 14), and complexity of care in ICU/isolation (7.5%, n = 3). Patients were treated by the PCCS according to their actual needs and under the consideration of our treatment algorithm [15]. Variations among the participants regarding their need for supportive care are also apparent in days of PCCS co-care, which ranged between 1 and 245 days (29 ± 41.18). There were 36 patients suffering from oncological disease (90%), of whom 29 (80.6%) had advanced metastatic disease. Among all included non-malignant patients, two (5%) were affected by short bowel syndrome, one (2.5%) suffered from complicated septicemia, and one (2.5%) participant was critically ill due to cardiac decompensation related to congenital heart failure. Some of our patients (17.5%, n = 7)) were isolated to avoid infection by hospital staff or visitors due to infectious pathogens or as part of a reverse isolation program. There were 37 patients (92.5%) receiving long-acting analgesics, of whom 6 patients (15%) were treated with non-opioid analgesics such as non-steroidal anti-inflammatory drugs or paracetamol with or without adjuvants, 1 patient (2.5%) received weak opioids with non-opioid analgesics, and the majority (n = 30, 75%) required potent opioids with µ-receptor activity with or without non-opioid analgesics and with or without adjuvants. Despite pain, 3 patients (7.5%) did not receive any regular analgesics due to their personal choice. Mean Karnofsky score was 45.5 ± 14.97 while mean health-related quality of life measured by EQ-5D-5 L was 42.0 ± 23.08. Values less than 50 in both assessment instruments indicate that patients included within this study were in an overall substantially disease-burdened condition. There were no significant differences in gender or age for any of the following collected data. Inpatient characteristics are given in Table 1.

**Table 1:** Inpatient characteristics, main diagnosis, and health-related quality of life by EuroQol 5 Dimension 5 Level; (n = 40) Abbreviations: SD = Standard deviation; Q1 = First quartile; Q3 = Third quartile; EQ-5D-5 L = EuroQol 5 Dimension 5 Level; PC = Palliative care; VR = Virtual reality

Age (years) mean (SD) median (Q1, Q3; range)	51.9 (15.81) 54 (39, 64; 19–80)
Karnofsky performance status scale mean (SD) median (Q1, Q3; range)	45.5 (14.97) 50.0 (40.0, 50.0; 10–80)
health-related quality of life (EQ-5D-5 L) mean (SD) median (Q1, Q3; range)	41.9 (23.08) 43 (20, 60; 0–95)
Female sex, n (%) Patients receiving specialized PC, n (%) Patients receiving usual PC, n (%)	21 (52.5%) 37 (92.5%) 3 (7.5%)
Cancer diagnosis, n (%)	36 (90%)
Isolation, n (%)	7 (17.5%)
Prior experience with VR, n (%)	3 (7.5%)

### Procedural characteristics

While there were only three patients with previous experience of VR (7.5%), there were five (12.5%) with general knowledge of its use in healthcare and the treatment of patients.

On average, each session lasted 31.3 ± 11.16 min. There were several video experiences patients could choose from; one person watched a single video (2.4%), five of them watched two videos (14.6%), eight watched a number of three videos (19.5%), and there were 26 participants who watched more than three videos (63.4%). Among all videos, the most viewed ones were Iceland (75% of all participants, n = 30)), London (62.5%, n = 25), Dolphins (57.5%, n = 23)), Dream Beach (47.5%, n = 19), and Wild West (42.5%, n = 17). In addition, games were played by 20% (n = 8) of the patients. The supervising medical student observed signs of physical relaxation during most of the interventions (e.g., relaxed muscles, smiles, expressions of joy), as well as numerous head movements. No withdrawals were seen within the study and no participant requested discontinuation of the VR intervention.

**Fig. 2:**
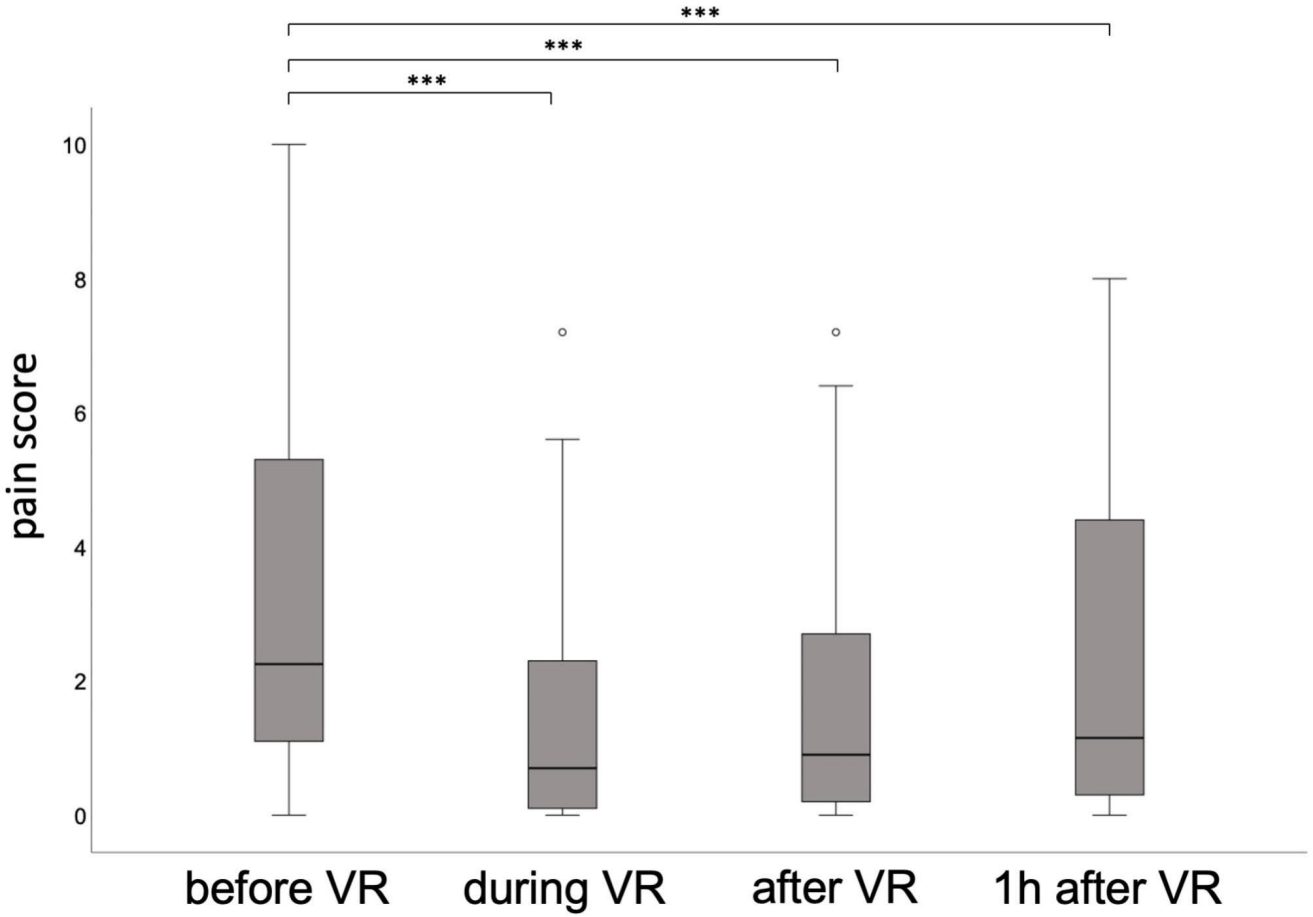
Pain boxplots score according to the timing of virtual reality intervention. *Legend: VR = Virtual reality; pain score = measured pain by Visual analogue scale (VAS)*

On average, isolated patients reported less pain compared to non-isolated patients prior to their VR intervention (isolated: mean VAS at baseline 2.3 ± 2.11, non-isolated 3.4 ± 2.76 p = 0.309). Pain reduction was fairly similar across both groups. Significant pain reduction (p-values ≤ 0.05) was observed for all three measuring points (during, immediately after, and one hour after the VR intervention) as compared to the baseline values (Fig. 2).

Overall, there was only one significant variation when comparing the Pain Out questionnaires. Sleep impairment caused by pain was answered significantly differently pre- and post-intervention (mean NRS at baseline 4.7 ± 3.23, mean NRS post-intervention 3.6 ± 3.05, p = 0.006). In total, there were seven patients (17,5%) who reported lower values than before. All other values had no significant variation.

Most participants described a feeling of joy or happiness following the VR intervention (82.5%, n = 33). Nevertheless, there were two participants reporting sadness (5%) due to reminiscences and wishes to be healthy again so they are able to experience the seen things on a more realistic basis.

No serious side effects occurred. However, after the intervention, three patients reported either nausea or dry eyes (7%), two reported headache (5%), while no one reported dizziness.

### Evaluation of individual patients’ experience

Following the intervention, all patients were asked to rate their VR experience as very good, good, fair, or poor. The responses are shown in Table 2. We compared these ratings by gender but did not detect any differences (chi-square test, p = 0.165).

**Table 2:** Evaluation of the virtual reality intervention by the patients. Participants could choose between four different response options

	female	male	total	
	n	n	n	%
very good	14	7	21	52.5
good	5	8	13	32.5
fair	2	4	6	15
poor	0	0	0	0
total	21	19	40	100

A total of 82.5% (n = 33) would like to experience a VR session once again. A significant correlation (Spearman correlation 0.463, p = 0.003) was found regarding the evaluation of their VR experience and a request for further sessions, which means that the better the VR experience was evaluated, the greater was the request for further VR interventions.

## Discussion

We aimed to investigate whether VR might offer to be a beneficial supplementary and feasible tool for pain relief among patients receiving PC. To our knowledge, this study is one of the first to investigate the feasibility of VR for palliative care in Europe and the very first study on this topic conducted in Germany. As we live in pandemic times with restricted person-to-person contacts in many places, VR provides an opportunity for escaping isolation through distraction.

Highlight of this study lies in the application of VR technology, which has already been successfully used in several studies [21, 22, 27, 35–37]. Evidence is emerging that immersive VR experiences lead to stronger pain-relieving effects than non-immersive VR. Since we exclusively worked with immersive VR, we are unable to neither confirm nor disprove this hypothesis. In our study, significant pain reduction was shown to occur when using VR while, immediately after, and one hour after the VR intervention. Applicability was proven as well as high acceptance of the devices. Remarkably, the acceptance was totally unrelated to patient’s age.

Except rare reports of nausea, headache and dry eyes, there were no serious side effects. However, similar to Mosadeghi et al.‘s study, some participants requested improvements regarding weight reduction and a more comfortable fit of the VR devices. In addition, some participants reported intermittent difficulties to maintain concentration [27].

When comparing the Pain Out questionnaires, only one significant improvement (sleep impairment) was found. There were no significant deviations in any of the other values. A limiting factor in this context could be that this questionnaire was not originally developed for PC. Perhaps this questionnaire simply lacks relevant elements that would be appropriate for our study.

Recently, an Australian study demonstrated the feasibility and high acceptance of VR (Oculus Rift® with the non-interactive video Nature Trek®) for patients with pain caused by cancer in PC [38]. The same video was shown to a comparison group by using a 2D laptop display. Pain intensity decreased significantly during and immediately following both interventions, although there was no significant difference between 3D HMD VR and 2D screen. Not surprisingly, higher levels of presence occurred within the VR intervention group. However, the number of participants amounted of only 14 including one drop-out. Also Niki et al. investigated the applicability of VR treating 20 terminally ill cancer patients with similar improvements as reported by Austin et al. [38, 39].

Another study showed the efficacy of a single VR intervention (HMD VR with Ocean Rift® or sitting on the beach with the “Happy Track”) when combined with morphine versus treatments with solely morphine for the reduction of pain and anxiety among patients with breast cancer using a randomized control design [40]. Just a year later, these authors et al. performed a review and strongly recommended the use of VR for female cancer patients as a supplementary intervention for the treatment of pain and anxiety [41].

Even one hour after ending their VR intervention, a significant pain reduction was shown according to our results on the analgesic effects of VR. Short-term effects of VR seen in this study are most likely due to distraction. Presumably, attention is directed to the virtual world and less perception of pain occurs. Besides this, also longer-lasting effects are discussed as a result of neuromodulation. Thus, a functional magnetic resonance imaging (fMRI) study by Álvarez-Pérez et al. revealed decreased activity in brain areas associated with anxiety and pain after VR interventions [42]. In addition to distraction, Ahmadpour et al. mentioned shifting to virtual objects and skill building based on interaction as mechanisms of VR’s analgesic effects [43].

As a potential limitation, in view that the investigators monitored all interventions, we cannot exclude investigator bias. This may have confounded the study outcome, for instance by affecting well-being and the perception of subjective pain. However, this is most likely a systemic bias, since all participants received increased attention throughout the study. Also, the “VAS questionnaire” was self-constructed and is not standardized, although visual analog scales are proven and validated instruments for the measurement of individual pain over several years [44].

Each participant was invited to choose the number and type of videos or games independently, as well as duration of the intervention was arbitrary, according to patients’ preferences and capabilities. There is need for further randomized clinical trials to investigate correlations between the duration of VR use and its benefits. Since there are many outpatients requiring PC, further studies should also evaluate the use of VR across these settings. We assume a wide range of different usages, considering that mobility of the cervical spine is necessary for the complete experience of using and viewing VR. While patients with high mobility of the head and cervical spine are able to fully utilize the device, those patients who are immobile may potentially strongly benefit from VR technology as a result of newfound freedom of movement in virtual worlds. Yet, it is important to consider that head and neck cancer with large tumor mass could limit the use of VR technology particularly in lower middle-income countries. In this context, it is important to be aware that using VR equipment could even cause pain instead of relieving it.

Currently, VR technology and its use in PC is quite unfamiliar. Nevertheless, presented data indicate an enormous potential for PC and suggests further studies to optimize this technology for individual needs. For example, personalized film recordings to support autobiographical work involving patients might offer an address for research. Among other imaginable aspects, it would be also useful in the context of patients’ desire to revisit certain places.

Based on the benefits of dignity therapy in PC [9, 45], VR interventions may be interpreted as a modern version to support patients’ well-being and sense of dignity. In their review, Rodríguez-Prat et al. exemplified the way dignity and autonomy are intertwined [46]. In particular, disabled patients and those suffering movement restrictions due to illness benefited tremendously through VR technology. For example, patients were able to experience virtual excursions to the countryside, to visit popular cities, or places of personal interest. This might help to strengthen patients’ sense of autonomy and provide an entry point into conversations.

We launched our study in 2018, and even then, VR devices were affordable (usable models started around $100–200). Increasing availability and decreasing costs will enable researchers and clinicians to conduct further studies more easily. Indeed, there is a realistic opportunity for patients to independently incorporate VR into pain management even now, based on manageable costs as well as it’s not pharmacy-only. Thus, in certain cases, physicians may recommend its use to patients even at this early state of research.

## Conclusion

Overall, we demonstrated that a single VR experience is an effective non-pharmacological treatment for the relief of pain in PC. There is broad acceptance and high potential for its future use in PC. Notably, its use is appropriate for PC due to its ability to encompass the approach of a total pain and symptom therapy, to enhance patients’ dignity and autonomy, and to open up conversations and foster optimism by providing content that differs from daily reality. Future research ought to include if and to what extent VR could reduce the necessity of pharmacological pain relief and potentially be applied to larger cohorts within the health care system. Since there is a solid foundation of positively evaluated evidence-our study also reinforces its benefits-available, including VR in guidelines for pain management and PC merits consideration.

## Data Availability

The datasets used and/or analyzed during the current study are available from the corresponding author on reasonable request.

## List of abbreviations

**PC**: Palliative care.
**VR**: Virtual reality.
**VAS**: Visual analogue scale.
**HRQOL**: Health-related quality of life.
**EQ-5D-5L**: EuroQol 5 Dimension 5 Level.
**SD**: Standard deviation.
**PCCS**: Palliative care consultation service.
**NRS**: Numeric rating scale.
